# Perivascular adipose tissue in autoimmune rheumatic diseases

**DOI:** 10.1016/j.phrs.2022.106354

**Published:** 2022-07-14

**Authors:** Hong Shi, Hanping Wu, Michael A. Winkler, Eric J Belin de Chantemèle, Richard Lee, Ha Won Kim, Neal L. Weintraub

**Affiliations:** a Division of Rheumatology, Medical College of Georgia at Augusta University, Augusta, GA, USA; b Division of Cardiology, Department of Medicine, Medical College of Georgia at Augusta University, Augusta, GA, USA; c Department of Radiology and Imaging, Medical College of Georgia at Augusta University, Augusta, GA, USA; d Department of Surgery, Medical College of Georgia at Augusta University, Augusta, GA, USA; e Vascular Biology Center, Medical College of Georgia at Augusta University, Augusta, GA, USA

**Keywords:** Perivascular adipose tissue, Cardiovascular disease, Autoimmune rheumatic disease, Inflammation

## Abstract

Perivascular adipose tissue (PVAT) resides at the outermost boundary of the vascular wall, surrounding most conduit blood vessels, except for the cerebral vessels, in humans. A growing body of evidence suggests that inflammation localized within PVAT may contribute to the pathogenesis of cardiovascular disease (CVD). Patients with autoimmune rheumatic diseases (ARDs), e.g., systemic lupus erythematosus (SLE), rheumatoid arthritis (RA), psoriasis, etc., exhibit heightened systemic inflammation and are at increased risk for CVD. Data from clinical studies in patients with ARDs support a linkage between dysfunctional adipose tissue, and PVAT in particular, in disease pathogenesis. Here, we review the data linking PVAT to the pathogenesis of CVD in patients with ARDs, focusing on the role of novel PVAT imaging techniques in defining disease risk and responses to biological therapies.

## 1. Introduction

Autoimmune rheumatic diseases (ARDs), including systemic lupus erythematosus (SLE), rheumatoid arthritis (RA), psoriasis and psoriatic arthritis (PsA), systemic sclerosis (SSc) and vasculitis, are associated with an increased risk of cardiovascular disease (CVD) which cannot be explained solely by traditional risk factors [1]. Endothelial dysfunction and premature atherosclerosis are commonly seen in these patients, suggesting that ARDs may work in concert with traditional risk factors to promote CVD [2]. The prevailing theory of CVD pathogenesis is that chronic low-grade systemic inflammation leads to prolonged endothelial activation, accompanied by an excessive production of pro-thrombotic/pro-coagulants and autoantibodies [3]. However, the underlying mechanisms leading to such an increased CVD risk in ARDs are poorly understood.

Inflammation is a central factor in both atherosclerosis and ARDs and is a key element in the pathogenesis of vulnerable plaque and vascular calcifications [4,5]. Both innate and adaptive immune systems are implicated in a complex network of molecular and cellular inflammatory interactions at the site of atherosclerotic lesions [6]. Systemic markers such as high sensitivity C-reactive protein (hsCRP), and pro-inflammatory cytokines such as tumor necrosis factor (TNF)α and interleukin (IL)− 6, have been widely used as clinical markers of CVD prediction [7] and may have a mechanistic role in CVD associated with ARDs. Quantifying the extent of vascular inflammation is emerging as a powerful approach to predicting cardiovascular events and could lead to the development of new therapeutic targets for CVD associated with ARDs.

Perivascular adipose tissue (PVAT) resides at the outermost boundary of the vascular wall, surrounding most conduit blood vessels, except for the cerebral vessels, in humans [8]. In small and micro-vessels, PVAT is an integral part of the vascular wall itself, while in large vessels, PVAT is contiguous with the adventitial layer. Beyond providing a structural support to blood vessels, PVAT is recognized as metabolically active tissue with distinct endocrine/paracrine functions that regulates vascular homeostasis by secreting biologically active substances such as adipokines, cytokines, extracellular vesicles and others [8]. Given its proximity to the vascular wall, and the lack of a structural barrier, PVAT is well positioned to interact directly with the vasculature in a bidirectional manner. PVAT is a rich source of both pro- and anti-inflammatory mediators, and in pathological states such as obesity, PVAT displays a “dysfunctional” phenotype, characterized by profound immune infiltration, imbalance of lipid accumulation and clearance, as well as enhanced expression of inflammatory cytokines/adipokines and increased oxidative stress, thus promoting local endothelial dysfunction and atherosclerosis [9,10]. Accumulating data from both human and experimental animal models suggests that dysfunctional PVAT is involved in atherosclerosis, hypertension, obesity, aneurysms and thrombosis. However, the role of PVAT in the pathogenesis of CVD in the setting of ARDs has received little attention.

In this review, we will first briefly summarize the general features of PVAT, its effects on vascular function and its contribution to CVD. Next, we will summarize the imaging features of PVAT with an emphasis on clinical importance and diagnostic values. Subsequently, we will elaborate on the potential role of PVAT in different ARDs.

## 2. PVAT pathophysiology

### 2.1. General features of PVAT

Phenotype and function of PVAT vary depending on species and anatomic location, displaying features of white adipose tissue (WAT), brown adipose tissue (BAT)/”beige” fat, or an intermediate between the latter two. Epicardial adipose tissue (EAT), which is partly contiguous with PVAT, is located between the inner layer of the pericardium and the free wall of the ventricle. A large portion of EAT extending from the surface of the heart to the adventitia of the coronary arteries is called the peri-coronary adipose tissue (PCAT), a predominantly WAT depot. EAT/PCAT volume measurement is considered a surrogate indicator of metabolic syndrome and has been used for cardiovascular risk stratification [11,12]. Intriguingly, increased EAT volumes have also been observed in SLE patients [13]. Thoracic aortic PVAT (tPVAT) contains beige fat and mesenteric arterial PVAT (mPVAT) resembles WAT, while abdominal aortic PVAT (aPVAT) displays a mixed phenotype [14].

### 2.2. PVAT and vascular tone

PVAT regulates vascular tone in both contractile and anticontractile fashion by PVAT-derived factors (e.g., adipokines, cytokines and growth factors) that target endothelial cells and vascular smooth muscle cells (VSMC). PVAT-derived relaxing factors (PVRFs) include leptin and adiponectin (APN) [15], apelin [16]prostaglandins [17], nitric oxide (NO) [18], hydrogen sulfide [19], hydrogen peroxide [18,20], and angiotensin 1–7 [21], which modulate vascular tone through endothelium-dependent and endothelium-independent mechanisms [22]. In addition to PVRFs, PVAT can also release PVAT-derived contracting factors (PVCFs), including norepinephrine [23], chemerin [24], and reactive oxygen species [25] which augment vasoconstriction through endothelium-dependent and -independent effects. PVAT may also play an important supportive role in dampening mechanical forces transmitted to coronary arteries during the cardiac cycle. Interestingly, PVAT was reported to preserve vasomotor function in human saphenous vein grafts employed during coronary bypass surgery [26], which is also consistent with a supportive role. Additionally, PVAT-derived adipokines can diffuse through the vascular wall, enter the vascular lumen and access the downstream microcirculation, which affords PVAT the ability to orchestrate vascular homeostasis [27] and facilitate insulin-mediated vasoreactivity and glucose uptake in tissue beds [28]. Whether adipokines derived from healthy PVAT might play a beneficial role to control ARDs is unknown. PVAT is necessary to maintain vascular homeostasis not only through endocrine/paracrine ways, but also via mechanical support for the vessel.

### 2.3. PVAT and vascular inflammation

PVAT releases an impressive repertoire of biologically active mediators that regulate vascular pathophysiology in both protective and detrimental ways. In healthy, lean states, PVAT can inhibit atherosclerosis by its thermogenic and fatty acid-scavenging properties, in keeping with the preponderance of brown/beige adipocytes. Under normal physiological conditions, PVAT secretes primarily anti-inflammatory molecules such as APN [29–31], omentin [32], interleukin (IL)− 10 [33–36], NO [37,38] and fibroblast growth factor-21 [39]. By contrast, in pathological settings such as obesity, white adipocytes predominate in PVAT, resulting in decreased clearance of plasma lipids and vascular mitochondrial dysfunction. In these conditions, PVAT predominately releases pro-inflammatory adipocytokines such as leptin [29,40], visfatin [41–44], chemerin [44], resistin [41,43,45], apelin [46], TNFα, monocyte chemoattractant protein-1 (MCP-1 or CCL2), IL-1β, IL-6, IL-8 and RANTEs (CCL5), in conjunction with enhanced infiltration of immune cells [29,47–50] and this may in turn aggravate vascular disease [51–54]. Genome-wide expression analyses of in vitro differentiated adipocytes isolated from PVAT surrounding human coronary arteries demonstrated that perivascular adipocytes have the potential to significantly modulate vascular inflammatory crosstalk in the setting of atherosclerosis by signaling both endothelial and inflammatory cells [55]. Decreased APN and enhanced IL-6, TNFα and toll-like receptor 4 expression in EAT is strongly linked to coronary artery disease (CAD), while increased APN has an atheroprotective effect by inhibiting macrophage-mediated inflammation [56]. Importantly, PVAT harvested from high-fat diet fed mice and transplanted to the abdominal aorta of lean recipient mice produced endothelial dysfunction and inflammation remotely in the thoracic aorta, suggesting that dysfunctional PVAT may contribute to vascular disease via systemic paracrine mechanisms [31].

### 2.4. PVAT, vascular remodeling and atherosclerosis

Healthy PVAT can oppose vascular remodeling and atherosclerosis by releasing factors that inhibit inflammation, proliferation and vaso-constriction. Low APN levels are positively associated with coronary artery disease [57]. In healthy mice, PVAT releases APN, which can protect against neointima formation after angioplasty [58]. High fat fed apolipoprotein E knockout mice lacking PVAT after deletion of peroxisome proliferator-activated receptor-γ in smooth muscle cells exhibited enhanced atherosclerosis when housed at 16 °C, consistent with anti-atherogenic properties of BAT [59]. However, under thermoneutral conditions, PVAT can adopt a dysfunctional, inflamed, WAT phenotype, contributing to vascular remodeling and atherosclerosis through a multitude of reported mechanisms, including PVAT-derived adipocytokines and extracellular vesicles, and dysregulation of progenitor cells [60,61].

In humans and animal models, PVAT-derived visfatin [42,62–64], resistin [65,66], leptin [67–69], chemerin [70–73], TNFα and MCP-1 [74,75] induce migration and/or proliferation in endothelial cells and VSMC. In contrast, APN and adrenomedullin inhibit endothelial cell and VSMC proliferation and migration [76–79]. Furthermore, PVAT-derived adipokines play a role in regulating fibroblasts. APN inhibits the expression of inducible NO synthase and migration of adventitial fibroblasts [80]. In contrast, visfatin induces the proliferation of cardiac fibroblasts and upregulates the expression of endothelial fibroblast growth factor in human endothelial cells [81,82].

## 3. Sex differences of PVAT

Accumulating clinical and experimental data suggest important sex differences in PVAT-dependent regulation of CVD. In the Framingham Heart Study, women were more likely to have a higher volume of thoracic PVAT compared to men [83]. Furthermore, the prevalence of excess thoracic PVAT increased by 50% with aging in women but only by 20% in men [83], suggesting that sex differences in PVAT distribution and localization may be an important contributor to sex-dependent changes in vascular pathophysiology. Ahmad et al. demonstrated that, in porcine coronary arteries, PVAT mediates anti-contractile activity in healthy, young female, but not male, animals [84]. Moreover, Small et al. reported that PVAT from hypertensive female rats retains more anti-contractile capacity than that from male rats [85]. In contrast, Watts et al., showed that, in the setting of high fat diet, anti-contractile function of PVAT is lower in female Dahl salt-sensitive hypertensive rats compared to males [86]. These divergent results suggest that sex differences in PVAT function may vary depending upon the species, strain, and experimental model.

## 4. Imaging features of PVAT

Imaging PVAT may help in assessment and therapeutic management of patients with diverse forms of CVD, and in characterizing animal models of vascular disease. Temporal and spatial development of aortic PVAT and luminal plaque in mice can be imaged by micro computed tomography (CT), carotid ultrasound, echocardiography and magnetic resonance imaging (MRI) [87].

Ultrasound vascular indexes such as carotid intima-media thickness (cIMT) and epicardial fat thickness (EFT) have been widely employed to assess atherosclerotic disease [88]. Recently, a new vascular index, the periarterial adipose tissue intima media adventitia (PATIMA) index, has been introduced as a combination of arterial wall and adipose tissue indices [cIMT, carotid extra media thickness (cEMT), EFT and body mass index (BMI)]. PATIMA index correlated with the presence and the severity of CAD [89–91], and more complex CAD in high and very-high CV risk patients [90]. In addition, the PATIMA index and carotid vascular indices (cIMT, cEMT) predicted coronary revascularization in patients with high or very high CV risk [92].

**Fig. 1.**
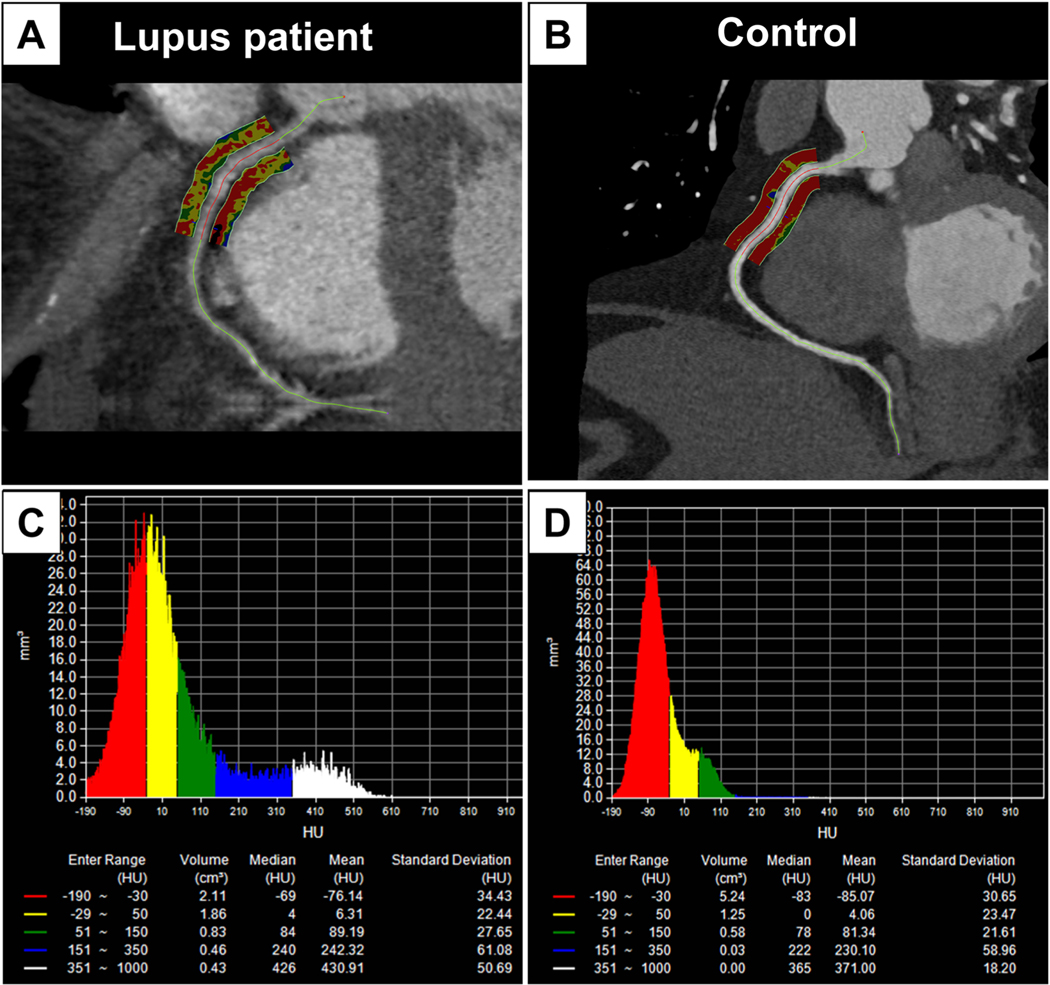
Representative images of FAIPVAT color maps around right coronary artery in a lupus (A) and control (B) patient (unpublished data). The perivascular adipose tissue attenuation index (PAAI) was sampled in 3-dimensional layers of 5 mm from the outer vessel wall of RCA. (C, D) Histograms of voxel computed tomography (CT) attenuations within the volume of interest. The mean CT attenuation within the range between − 190 and − 30 HU, which was defined as PAAI, was − 76.14 HU in the lupus patient (C) and − 85.07 HU in the control patient (D).

MRI has been regarded as the gold standard for estimation of whole-body adipose tissue [93]. Cardiac MR (CMR) can volumetrically measure EAT volume and has been proven to be feasible and reproducible for the assessment of EAT [94]. However, CMR is not used as widely as CT due to its relatively limited clinical availability and incompatibility with implanted metallic objects. Due to its high spatial resolution and three-dimensional views, cardiac CT is current the preferred method for EAT assessment over echocardiography and CMR [95]. Antonopoulous et al. proposed the fat attenuation index (FAI) as a new biomarker to explore the regional biological variability of coronary artery PVAT [96]. In this translational study, PVAT around the epicardial coronary arteries was defined as the adipose tissue located within a radial distance from the outer vessel wall equal to the diameter of the adjacent coronary vessel. In the presence of normal PVAT with large, mature, lipid-laden adipocytes, PVAT attenuation was lower, while the PVAT attenuation was increased when vascular inflammation resulted in lipolysis with smaller adipocytes containing fewer lipid droplets. By analyzing the changes of attenuation within PVAT, the inflammatory burden of the adjacent coronary artery could be estimated. This information was mapped using AI-enhanced algorithms to measure the weighted attenuation shifts within PVAT. This technique can be readily applied to assess coronary PVAT inflammation in patients with ARDs, such as SLE, as shown in Fig. 1 (unpublished data).

In contrast, micro-CT lacks sufficient resolution to accurately quantify PVAT inflammation in mice, given the small size of vasculature and the fast motion velocities. However, newly developed contrast agents such as poly-coated gadolinium nanoparticles can significantly improve the quality of images and may facilitate more detailed vascular imaging studies in the future [97].

## 5. PVAT in autoimmune rheumatic diseases

### 5.1. PVAT in SLE

SLE is a heterogeneous systemic inflammatory autoimmune disorder that primarily affects women of childbearing age, characterized by profound dysregulation of immune responses and multiorgan involvement with a high risk for CVD due to accelerated atherosclerosis, which cannot be explained by traditional risk factors or SLE-specific attributes [98–100]. Adipose tissue inflammation has been suggested to play an active role in atherosclerosis in SLE [101]. Prior studies have reported increased levels of pro-inflammatory adipocytokines in serum of SLE patients [102–107], and elevated leptin levels are associated with an increased risk of atherosclerosis in lupus patients [107]. Moreover, leptin treatment further enhanced endothelial dysfunction and atherosclerotic lesions in lupus-prone mice fed a high fat diet [108]. While these studies support a general role for pro-inflammatory adipocytokines as a source of chronic inflammation, the specific adipose tissue depots contributing to vascular damage in SLE have not been identified. Lipson et al. reported that EAT volume was greater in patients with SLE than controls. Within SLE patients, after adjusting for age, race, sex, and waist circumference, EAT volume was associated with cumulative corticosteroid dose, current corticosteroid use, HDL cholesterol, and triglycerides. Furthermore, EAT was significantly correlated with coronary artery calcium (CAC) score, but the association was attenuated after adjustment for Framingham risk score. These findings suggest both traditional risk factors and SLE therapies likely contribute to the pathogenesis of CVD in SLE patients [109].

**Fig. 2.**
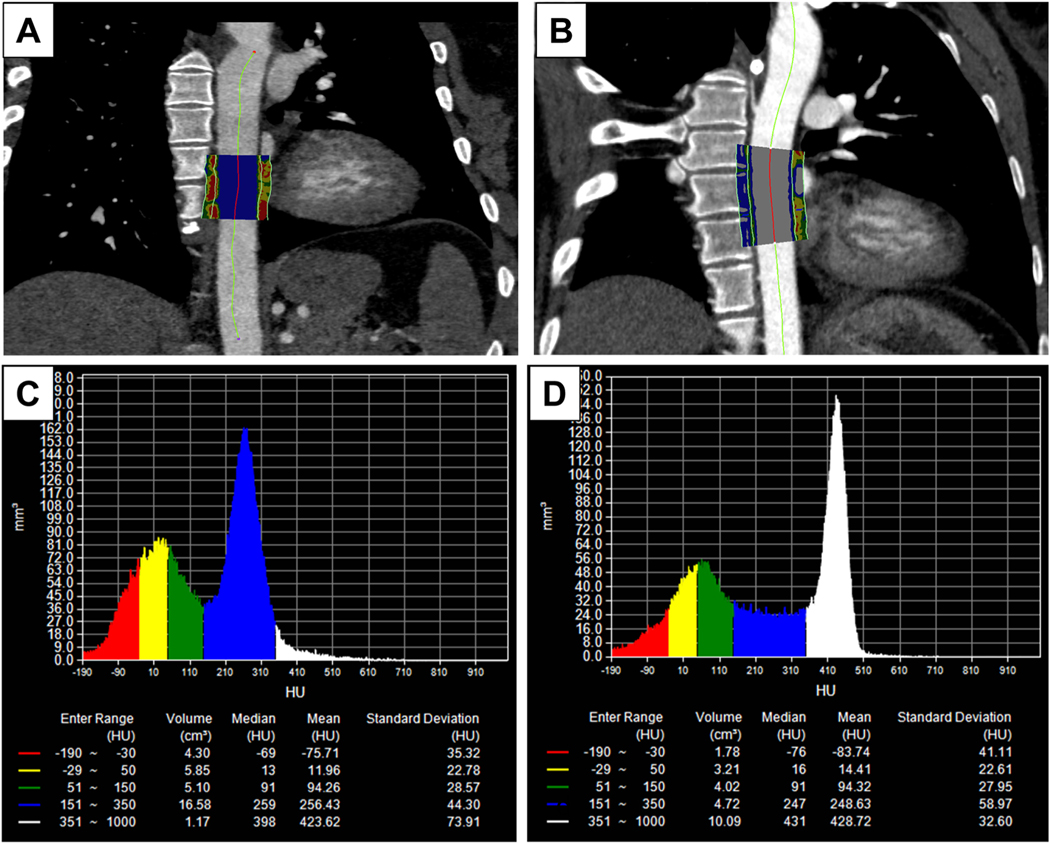
Representative images of FAIPVAT color maps around descending aorta in a lupus (A) and control (B) patient (unpublished data). The periaortic adipose tissue attenuation index (PAAI) was sampled in 3-dimensional layers of 5 mm from the outer vessel wall of descending aorta. (C, D) Histograms of voxel computed tomography (CT) attenuations within the volume of interest. The mean CT attenuation within the range between − 190 and − 30 HU, which was defined as PAAI, was − 75.71 HU in the lupus patient (C) and − 83.74 HU in the control patient (D).

Recently, women with SLE were noted to have higher volumes and densities (determined by CT scan) of PVAT surrounding the thoracic aorta compared to heathy control subjects [110,111]. Denser PVAT was associated with PVAT volume and aortic calcification [111]. In addition, PVAT inflammation was strongly associated with aortic calcification score in SLE patients independent of age, circulating inflammatory markers, CVD risk factors and BMI [111], which may be indicative of adipose dysfunction in PVAT of SLE patients. An example of a CT scan of normal control patient and a patient with SLE exhibiting PVAT inflammation of the descending aorta are shown in Fig. 2 (unpublished data). Collectively, these findings suggest that patients with SLE exhibit dysfunctional PVAT, which may contribute to local vascular pathology and CVD in SLE.

### 5.2. PVAT in RA

Rheumatoid arthritis (RA), the most common rheumatic disease, is also associated with elevated cardiovascular risk, as 50% of RA mortality is attributed to CVD[112]. Increased fat mass, altered fat/lean body composition and higher levels of pro-inflammatory adipokines such as leptin are linked to common CVD risk factors such as hypertension, hyperlipidemia, insulin resistance and metabolic syndrome in patients with RA [113,114]. Such risk factors are also associated with reduced efficacy of RA treatment [115]. Currently, there is no definitive evidence showing how PVAT inflammation affects the vascular wall in RA patients. Several studies have shown that EAT thickness is increased in patients with RA [116–119], and disease activity was independently associated with EAT thickness [119]. Furthermore, patients treated with TNFα inhibitors exhibited significantly lower EAT thickness than those treated with non-biological disease-modifying antirheumatic drugs [117]. EAT volume was associated with metabolic syndrome and cardiometabolic risk factors including insulin resistance, triglycerides, current smoking, and homocysteine levels [120]. The link between EAT volume, which correlates with FMD, cIMT and aortic pulse wave velocity [118,121], and coronary atherosclerosis/plaque morphology was investigated in patients with RA. EAT volume was strongly associated with plaque burden and vulnerability features in RA patients [122]. These findings suggest that EAT, and perhaps PVAT, may exacerbate the pathogenesis and severity of RA and early-stage atherosclerosis in RA patients.

Interestingly, a significant alteration in the aortic adventitia micro-environment was observed in CAD patients with RA. In CAD patients with or without inflammatory rheumatic disease who underwent coronary artery bypass graft (CABG) surgery, aortic tissue specimens were obtained to evaluate the association between RA and vascular inflammation. Patients with inflammatory rheumatic disease had more pronounced chronic inflammatory and mononuclear cell infiltration in the aortic media and inner adventitia compared to controls. Importantly, mononuclear cell infiltrates were detected in the tunica media only in those RA patients with inflammatory infiltration in the inner adventitia, which abuts PVAT. As PVAT inflammation has been linked to adventitial inflammation in prior studies, this finding may indicate a role for PVAT in driving the vascular wall inflammation through an outside to inside paracrine signaling manner [123]. In another study, the expression and extent of *pentraxin 3 (*PTX3), a molecule produced by various cells at sites of inflammation, was investigated in the outer layer of the aorta in RA patients with CAD. Interestingly, PTX3 was found to be expressed in endothelial cells and adipocytes, but not in infiltrating neutrophils or T cells, suggesting that inflamed adipocytes in PVAT may play an important role in the pathogenesis of RA-related CAD [124].

In a collagen-induced arthritis model of RA, contractile dysfunction was detected in the thoracic aorta [125]. Histologic analyses showed an increase in cell density and reduced vacuolarity in PVAT, without thickening of the aortic wall [126]. Additionally, leukocyte infiltration was detected in thoracic but not abdominal aorta. Furthermore, expression of galectin-3, a global marker of fibrosis, angiogenesis, inflammation and atherogenesis, was significantly higher in PVAT surrounding thoracic compared with abdominal aorta. Increased expression of CD11c, Arg1 and CD206 was also detected in thoracic PVAT in the setting of collagen-induced arthritis, while no significant changes were observed in abdominal PVAT. Collectively, these changes noted in PVAT in a mouse model of RA could be indicative of early vascular pathology and warrant further investigations, including translation to RA patients [126].

### 5.3. PVAT in psoriasis and psoriatic arthritis

Psoriasis and psoriatic arthritis are associated with an increased risk of CAD and major CV events associated with low-grade systemic inflammation [127–130]. Imaging studies in patients with psoriasis and psoriatic arthritis have shown signs of vascular inflammation [131]. A clinical study demonstrated that biological therapy reduced noncalcified coronary plaque burden in patients with moderate to severe psoriasis compared to patients with no biologic therapy [132,133].

Several studies have shown that EAT volume is increased [134–136] and correlates with cIMT [133,134] and CAC [132] in patients with psoriasis. In addition, males with psoriasis but no known coronary disease or diabetes had greater EAT volume than controls [137], suggesting that EAT volume may be an early indicator of psoriasis patients who are at increased risk for CVD.

Recently, perivascular FAI mapping was performed in a prospective cohort study of patients with moderate to severe psoriasis [138]. In this study, 134 patients who underwent coronary computed tomography angiography (CCTA) at baseline were divided into two groups according to whether they did or did not receive biologic therapy over the ensuing year. Among these patients, 82 received biologic therapy, while 52 did not. At baseline, 46 patients (27 in the treated group and 19 in the untreated group) had focal coronary atherosclerotic plaque, and the peri-coronary FAI was elevated in patients with moderate-to-severe treatment-naïve psoriasis. After biologic therapy, a significant decrease in FAI was observed at one year as compared to baseline, concurrent with improvement in skin disease. In contrast, no changes in FAI or skin disease status were noted in the untreated group. These findings suggest that perivascular FAI may be a useful marker to track the impact of therapies for rheumatic diseases on CAD risk [138].

Conversely, Bao et al. demonstrated that while psoriasis patients exhibited a higher atherosclerotic burden as quantified by the computed tomography-adapted Leaman score (CT-LeSc), and a higher prevalence of non-calcified plaques, their perivascular FAI was lower as compared with controls [139]. Therefore, the utility of perivascular FAI for evaluating coronary inflammation and CVD risk in patients with chronic low-grade inflammatory disease such as psoriasis requires further in-depth investigation [139].

### 5.4. PVAT in systemic sclerosis

Systemic sclerosis (SSc) is a chronic systemic disorder characterized by vasculopathy, organ fibrosis and immune dysfunction, and its prognosis depends on cardiopulmonary involvement [140]. The mortality ratio of SSc is estimated to be 3.5, with approximately one-third of deaths attributed to cardiac disease occurring more than one decade earlier than the general population [140–142]. Adipose tissue is a likely contributor to the pathogenesis of SSc, and in SSc patients, degradation of intradermal adipose tissue was reported to precede the onset of dermal fibrosis [143]. Recently, adipocytes adjacent to the wound have been shown to be reprogrammed to a myofibroblastic phenotype in a dynamic, reversible process termed adipocyte mesenchymal transition (AMT). AMT is mediated by lipolysis, which releases fatty acids that attract macrophages and influence vascularity [144]. However, inhibition of lipolysis in mature adipocytes produced an exacerbation of bleomycin-induced skin fibrosis [145]. Patients with SSc exhibit metabolic alterations in adipose depots as well as other tissues, including skin, lung, heart, liver and kidney [146–149]. Impaired mitochondrial beta-oxidation of fatty acids and excessive amino acid consumption were also observed in SSc patients [150,151]. Extracellular vesicles from adipose tissue ameliorated cardiac, lung and liver fibrosis in animal models of SSc, which suggest that the adipocyte secretome plays a central role in the pathogenesis of SSc [152].

SSc is an independent risk factor for increased coronary artery calcium deposition [153,154] and several studies have demonstrated a correlation between EAT and presence of SSc [155,156] EAT thickness was greater in SSc patients with no overt cardiac disease compared to matched healthy subjects, in association with increased inflammatory markers and metabolic risk factors [155]. Moreover, EAT volume was associated with the presence and severity of SSc, independent of CV risk factors and interstitial lung disease [156]. These findings suggest that EAT is a candidate for atherosclerosis risk assessment and might even contribute to the pathogenesis of vasculopathy in SSc patients.

Clinical studies showed that expression of endothelin, a vasoconstrictor produced by endothelial cells, is increased in SSc, suggesting that dysfunctional vascular endothelium may be associated with this disorder. However, the effect of PVAT-derived factors on endothelin expression and endothelial function in SSc patients is unknown. Adipose-derived stem cells (ASC) from SSc patients induced paracrine angiogenic effects on endothelial cells in vitro, but this effect was less potent compared to ASC derived from healthy donors [157]. Since the latter study did not specifically examine ASC derived from PVAT, further investigations are required specifically investigate the role of PVAT-derived paracrine factors in endothelial cell function in SSc patients.

### 5.5. PVAT in vasculitis

Vasculitis is a group of rare diseases that commonly involve inflammation of blood vessels, including giant cell arteritis (GCA) and/or polymyalgia rheumatica (PMR), Takayasu arteritis (TAK), and Kawasaki disease (KD). While multiple studies have demonstrated an association between adventitial/periadventitial tissues and vasculitis, direct evidence of a pathogenic role of PVAT has not been explored.

GCA is the most common systemic vasculitis and may involve the aorta, its branches, and smaller muscular vessels [158,159]. PMR is the second most common inflammatory rheumatic disease of the elderly after rheumatoid arthritis [160], and an association between GCA and PMR has been observed [161]. Temporal artery biopsy is considered the gold standard for the diagnosis of GCA, [162] with pathognomonic features of transmural active inflammation with or without giant cells involving the media [163,164]. However, a more limited inflammation, restricted to the adventitial and/or periadventitial tissue, has been observed in a group of patients with GCA/PMR [165,166], though the clinical significance of this finding remains controversial [167–169]. Recently, a retrospective cohort study was performed to evaluate the characteristics and significance of inflammation limited to the adventitial and/or periadventitial tissue of temporal arteries. The investigators found that inflammation restricted to this site had a high specificity and positive predictive value for GCA and/or PMR, with an almost 4-fold increase in likelihood of developing the disease [170]. Given that PVAT is juxtaposed to the adventitia, it is tempting to suggest a role for PVAT in promoting periadventitial inflammation in GCA/PMR patients.

TAK is a systemic large vessel vasculitis that typically affects young women and is characterized by granulomatous inflammation of all three layers of the aorta and its main branches, leading to fibrotic stenosis, occlusion, aneurysms, vessel wall thickening and accelerated atherosclerosis [171]. In the chronic phase, there is fibrotic replacement in the media and adventitia, extending into the vasa vasorum, along with intimal thickening. A high prevalence of metabolic syndrome was also observed in patients with TAK [172]. In addition, increased expression of pro-inflammatory cytokines such as interferon γ, IL-6, IL-12 and IL-17 has been reported in TAK. Interestingly, aberrant adipokine levels have also been reported in TAK, manifested by excessive secretion of leptin and visfatin and reduced APN [173–176]. Leptin level may in particular be a strong predictor of long-term progression of TAK arteritis [177]. Recently, arterial inflammation and atherosclerosis in TAK were assessed by quantifying PCAT and periaortic adipose tissue (PAAT) density on CCTA [178]. Patients with TAK had higher PCAT and PAAT densities on CCTA than patients with atherosclerosis, CAD or control subjects, independent of potential patient-level confounding factors. As compared with than PAAT, PCAT density was more closely linked with markers of clinical disease activity in TAK [178]. These data suggest that PVAT may not only play a role in the increased risk of CAD, but also in the pathogenesis of TAK itself.

KD is a systemic vasculitis syndrome of medium-sized arteries that occurs mostly in children, and coronary arteries are the most frequently involved blood vessels in KD [179]. Formation of coronary artery aneurysms with thrombotic occlusion was reported in a majority of autopsy cases involving KD [180,181]. In addition, dynamic infiltration of inflammatory cells has been detected in both coronary arteries and the aorta in KD [182]. The inflammatory cell infiltration involved the tunica intima and tunica adventitia on the 6th disease-day, reached all layers of blood vessels on the 13th disease-day, and peaked on the 18th disease-day. Then, inflammation gradually disappeared thereafter, and no significant infiltration was seen in the remote phase. Notably, adipokines have been suggested to play a role in the pathogenesis of KD. A meta-analysis showed that resistin levels were significantly elevated in KD patients, and KD patients with coronary artery lesions had higher serum resistin and adiponectin levels compared to KD patients without coronary artery lesions. Moreover, active KD patients had higher levels of resistin than the inactive group [183]. Future studies are required to investigate the mechanistic role of PVAT in regulating KD.

## 6. Perspectives

**Fig. 3.**
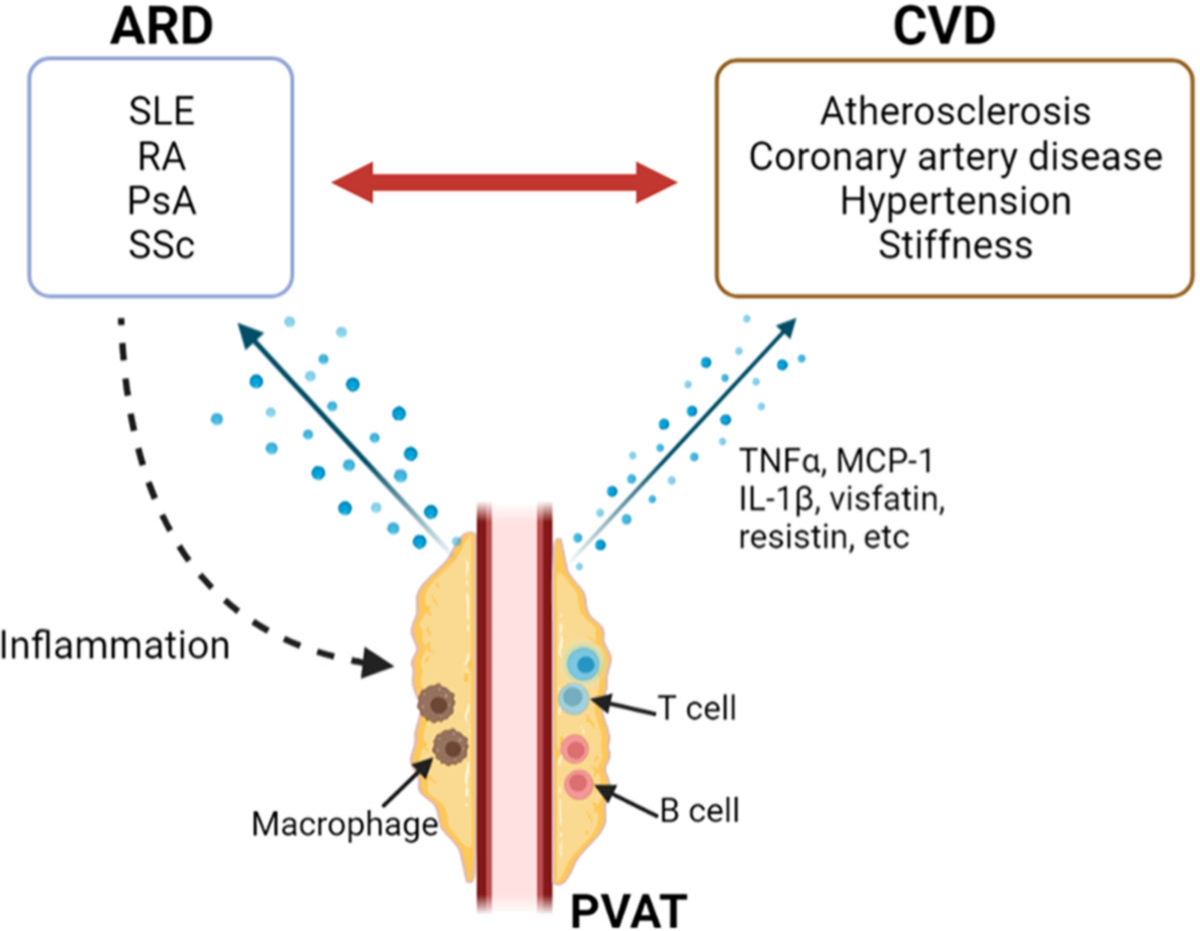
Potential interactive mechanisms whereby PVAT contributes to the pathogenesis of CVD in patients with ARD.

A growing body of data supports linkages between PVAT and ARDs. Chronic low grade systemic inflammation of ARDs could extend into the PVAT, inducing vascular dysfunction to contribute to CVD, with inflammatory crosstalk potentially promoting progression of the underlying ARD (Fig. 3). Monitoring the extent of PVAT inflammation using CT techniques holds promise for predicting CVD risk, and response to therapies, in patients with ARDs. The mechanisms underlying the interplay between PVAT and cardiovascular disease in ARDs remain to be established.

## Data Availability

No data was used for the research described in the article.

## Abbreviations:

AMT: adipocyte mesenchymal transition
APN: adiponectin
ARD: autoimmune rheumatic disease
BAT: brown adipose tissue
CT: computed tomography
CMR: Cardiac magnetic resonance
CVD: cardiovascular disease
cEMT: carotid extra media thickness
cIMT: carotid intima-media thickness
CAC: coronary artery calcium
CAD: coronary artery disease
EFT: epicardial fat thickness
FAI: fat attenuation index
GCA: giant cell arteritis
IL: interleukin
KD: Kawasaki disease
MRI: magnetic resonance imaging
mPVAT: mesenteric arterial PVAT
MCP-1: monocyte chemoattractant protein-1
NO: nitric oxide
PTX3: pentraxin 3
PAAT: periaortic adipose tissue
PCAT: peri-coronary adipose tissue
PVAT: Perivascular adipose tissue
PMR: polymyalgia rheumatica
PsA: psoriasis and psoriatic arthritis
PVRFs: PVAT-derived relaxing factors
RA: rheumatoid arthritis
SLE: systemic lupus erythematosus
SSc: systemic sclerosis
TAK: Takayasu arteritis
tPVAT: Thoracic aortic PVAT
TNF: tumor necrosis factor
VSMC: vascular smooth muscle cells
WAT: white adipose tissue
